# Personalized Treatment of Periodontitis in a Patient with McArdle's Disease: The Benefits from Probiotics

**DOI:** 10.1155/2023/5080384

**Published:** 2023-03-10

**Authors:** Salvatore Cannizzaro, Carolina Maiorani, Andrea Scribante, Andrea Butera

**Affiliations:** ^1^Ordinary Member of Academy of Advanced Technologies in Oral Hygiene Sciences, Siracusa, Italy; ^2^Unit of Dental Hygiene, Section of Dentistry, Department of Clinical, Surgical, Diagnostic and Pediatric Sciences, University of Pavia, Pavia 27100, Italy; ^3^Unit of Orthodontics and Pediatric Dentistry, Section of Dentistry, Department of Clinical, Surgical, Diagnostic and Pediatric Sciences, University of Pavia, Pavia 27100, Italy

## Abstract

**Introduction:**

McArdle's disease is a severe glycogen storage disease characterized by intolerance to exercise; patients have a syndrome of muscle intolerance to stress, associated with myalgia, cramps, fatigue, and muscle weakness. Periodontal disease is a multifactorial pathology of the supporting tissues of the teeth: one of the main factors is the formation of bacterial biofilm; its control favors the prevention and the maintenance of good health of the oral cavity; and some systemic pathologies can worsen the periodontal disease and hinder its therapy. This case report concerns a woman with McArdle's disease diagnosed with periodontal disease. *Material and Methods*. A 54-year-old female patient with McArdle's disease has been diagnosed with Stage 3 generalized periodontitis, Grade B. At the baseline, the patient had 82 pockets with probing pocket depth (PPD) equal to or greater than 4 mm. The patient was instructed in the correct methods of oral hygiene and was advised toothpaste and mouthwash based on probiotics; subsequently, a debridement was performed to remove etiological factors using Dental-Biofilm Detection Topographic Technique (D-BioTECH).

**Results:**

After 60 days, the number of pockets was reduced from 82 to 14 overall with PPD ≥ 4 mm and from 50 to 2 pockets with PPD ≥ 5 mm. Full mouth bleeding score (FMBS) increased from 48% to 15% and full mouth plaque score (FMPS) from 73% to 15%.

**Conclusions:**

In this case, the use of a correct brushing method combined with the D-BioTECH has reduced the disease state, with the use of probiotics at home to restore and maintain a healthy oral microbiome.

## 1. Introduction

Myophosphorylase deficiency (McArdle's disease), or glycogenosis type 5 (GSD5), is a severe glycogen storage disease (GSD) characterized by intolerance to exercise; patients have a syndrome of muscle intolerance to stress, associated with myalgia, cramps, fatigue, and muscle weakness [1]. Some studies reported that the prevalence is 1 in 100,000 (in the USA) and even rare in some European countries (1 in 170,000 in Spain and 1 in 350,000 in the Netherlands) [2–4]; however, it may be underestimated for causing mild symptoms. Another little-known topic is its association with periodontal disease. There are no studies in the literature that demonstrate a positive association or a predisposition of one of the two diseases towards the other. There are, however, studies that show that some GSDs predispose to periodontal problems [5, 6].

The oral microbiota can be classified into “core microbiota”, to which belong the species common to all healthy individuals, and “variable microbiota” composed, instead, of all those bacteria highly different from subject to subject, as they are more sensitive to external factors: among the most represented genera altogether, and therefore included in the core, *Streptococcus* spp., *Prevotella* spp., *Haemophilus* spp., *Rothia* spp., *Veillonellaceae* spp., *Neisseria* spp., *Fusobacterium* spp., and *Porphyrin* spp. are found [7, 8].

A well-balanced oral microbiota is fundamental in preventing the onset of pathologies of the oral cavity: diet, lifestyle, antibiotics, and social condition are the environmental factors that, more than any other, affect its composition and functionality; sugars, fats, and vitamins are among the most impactful nutrients in this type of microbiota [7].

Periodontal disease is a multifactorial pathology of the supporting tissues of the teeth: one of the main factors is the formation of bacterial biofilm; its control favors the prevention and maintenance of good health of the oral cavity [9].

With the increase in resistance to antibiotics and the desire to approach therapies defined as more “natural”, different treatments have been proposed compared to those focused on the use of the main antimicrobials for the elimination of bacterial species [10].

To date, the standard non-surgical treatment for periodontal disease involves professional scaling and root planing sessions, aimed at the removal of subgingival biofilm; additional therapies have been proposed and examined (glycine powders and/or erythritol for air-polishing and perio-polishing, laser, ozone, probiotics, chlorhexidine), for the evaluation of additional benefits to promote tissue healing and reduce the risk of bacteremia [11].

For the restoration of microbiological balance, the use of probiotics has been proposed to promote health-related bacterial growth and the use of probiotics, or microorganisms that, if administered in adequate quantities, can bring health benefits.

They are used, first of all, to antagonize dysbiosis by inhibiting the main periodontal pathogenic bacteria, thereby reducing the immunogenicity of the oral microbiota and modulating the inflammatory and immune response to reduce inflammation [12, 13].

In recent years, a different approach has been proposed for the non-surgical treatment of periodontal disease, namely Dental-Biofilm Detection Topographic Technique (D-BioTECH): the health professional must observe and share with the patient the topography of the bacterial biofilm, as evidenced by the use of the plaque detector, as sites most at risk of inflammation are visually intercepted, useful for motivational reinforcement of the patient, and improve the effectiveness of oral hygiene at home. It allows, in periodontal therapy, to have a minimally invasive clinical approach, because it allows the operator to instrumentate with polishing/air-polishing/selective mechanical instrumentation, exclusively following the topography of bacterial biofilm [14].

The purpose of this study is to describe the case of a patient with McArdle's pathology with periodontal disease treated by the clinical method D-BioTECH, and the use of probiotics to promote the reduction of the disease state and to reconstruct appropriate microbiological conditions for the health of the oral cavity.

## 2. Case Report

### 2.1. Diagnosis and Etiology

A 54-year-old female patient came to the observation in May 2022, complaining of gingival bleeding during brushing: the patient was unaware of her periodontal problems and did not have a regular professional oral hygiene session.

The medical history shows a familiarity with periodontal disease attributable to the father; the patient suffers from McArdle's disease, glycogenosis GSD V, due to the lack of the enzyme glycogen-phosphorylase muscle. McArdle is a serious disease, characterized by muscular intolerance to stress, associated with myalgia, cramps, fatigue, and muscle weakness [15]. McArdle sufferers experience pain or fatigue during some repetitive movements such as chewing [16] and washing their teeth [17]. About 33% of sufferers of this disease complain of a permanent weakness of the muscles closest to the trunk, such as the shoulder muscles [18]. The patient also says she needs many small snacks rich in complex carbohydrates.

**Figure 1:**
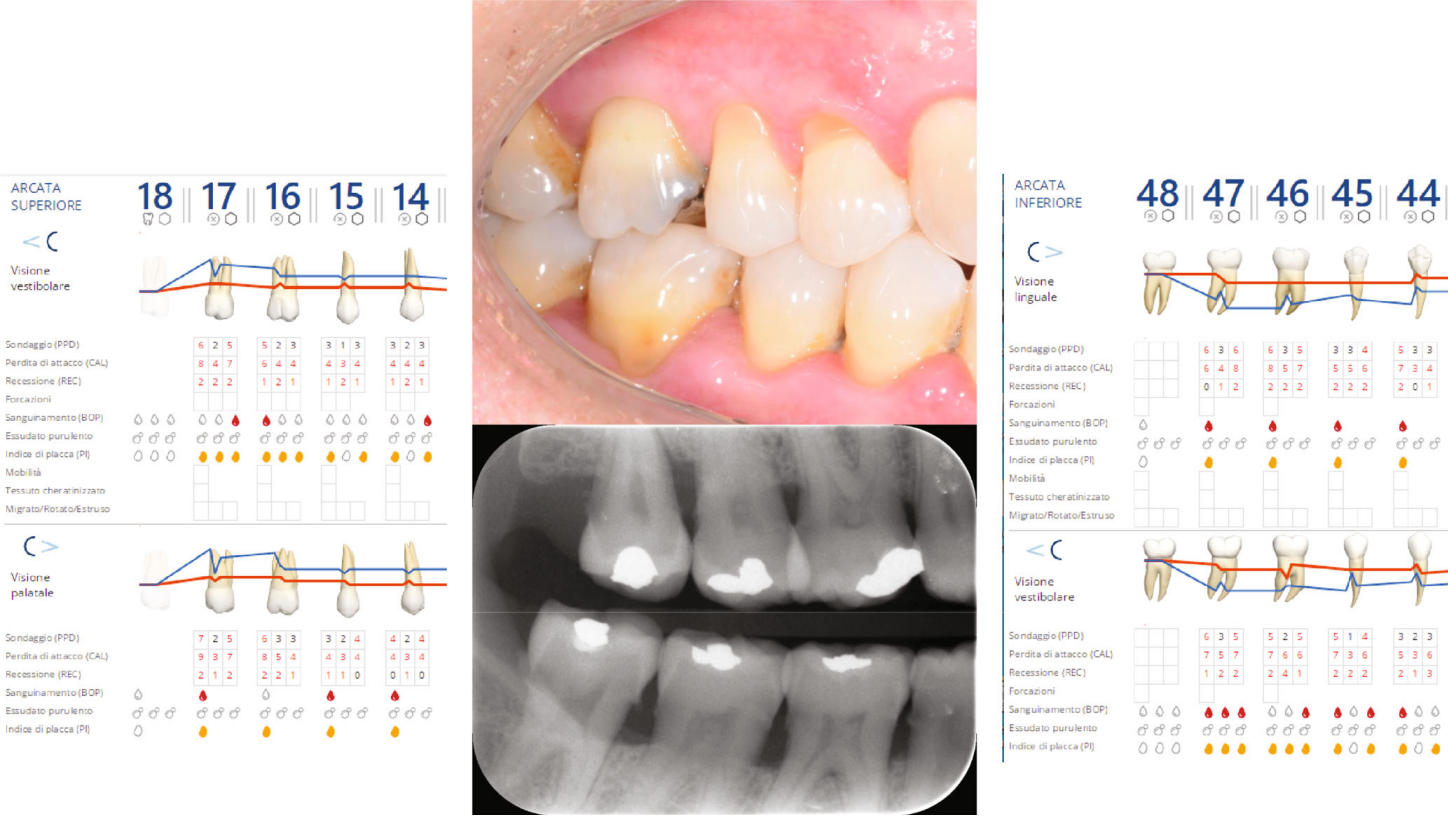
Baseline of the I and IV sextant: diagnostic deepening (photo, RX, and charting).

**Figure 2:**
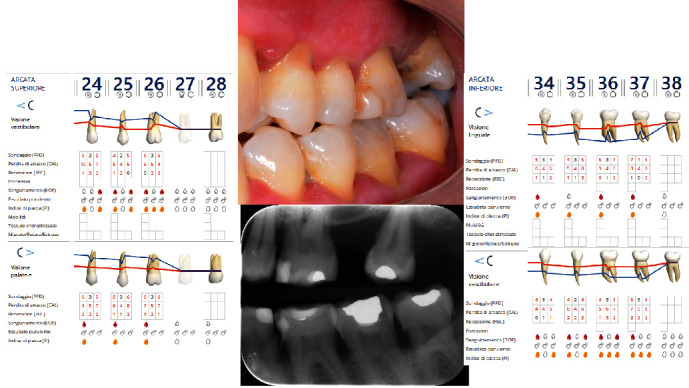
Baseline of the III and VI sextant: diagnostic deepening (photo, RX, and charting).

**Figure 3:**
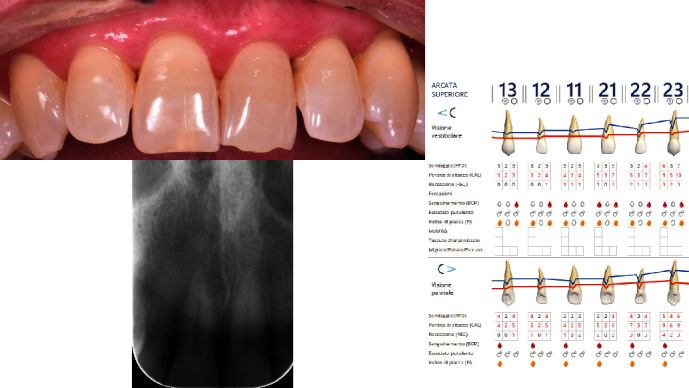
Baseline of the II sextant: diagnostic deepening (photo, RX, and charting).

**Figure 4:**
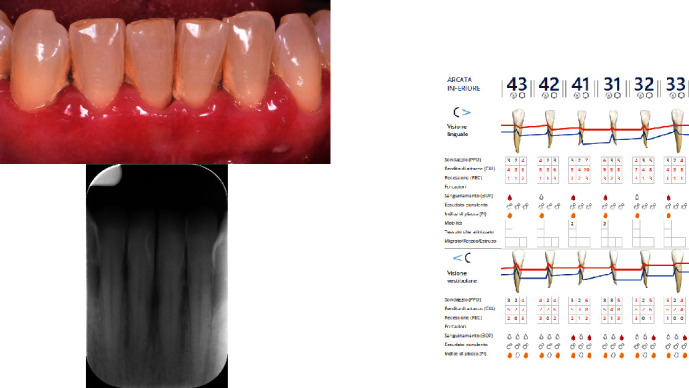
Baseline of the V sextant: diagnostic deepening (photo, RX, and charting).

The diagnosis made by the periodontist was Stage 3 generalized periodontitis, Grade B. At the baseline, the patient had 82 pockets with probing pocket depth (PPD) equal or greater than 4 mm: of these 82 pockets, 50 have PPD equal to or greater than 5 mm. The values of the inflammation indices are 48% for full mouth bleeding score (FMBS)and 73% for full mouth plaque score (FMPS). Figures 1, 2, 3, and 4 show periodontal charting, RX, and photos of the patient at baseline.

### 2.2. Treatment Objectives

When periodontal disease was intercepted, the patient was instructed and motivated by the “tailored brushing method” (TBM) approach, to promote correct lifestyles, reduce the greater number of periodontal pockets 5 mm, reduce FMBS and FMPS below 15%, and recreate a healthy oral microbiota with revaluation after 3 months [19].

### 2.3. Treatment

Once the periodontal disease was diagnosed, radiographic and photographic data were collected, and the periodontal clinical record and motivational interview (T0) were compiled. After 7 days, the patient was instructed to have an effective and efficient control of oral biofilm through the personalized and shared TBM approach. It was recommended to replace the manual toothbrush with a sonic electric toothbrush, which helps her with a minimal physical effort to improve her oral hygiene (DiamondClean 9000, Philips, Amsterdam, Netherlands).

The use of toothpaste with probiotics was suggested (Peribioma Pro Gengive Più, Coswell, Fano, Italy), which counteracts the symptoms and causes of periodontitis by promoting the rebalancing of the oral microbiota. The use of a mouthwash with probiotics (Treatment Mousse Peribioma, Coswell, Fano, Italy) was recommended twice a day for 1 minute without rinsing, drinking, and eating for at least the first 30 minutes [20]. For the cleaning of the interdental spaces, it was recommended to use antibacterial rubber brushes. To complete the oral hygiene at home, the importance of the use of chewable gum with probiotics (Peribioma Pro, Coswell, Fano, Italy) was explained. The latter are enriched with BIOMA microRepair (added with three billion specific probiotics), vitamin C, and D3, which help to protect the gums making them stronger and keeping the microbiota in balance even with a diet not properly correct [21].  Table 1 shows the compositions of the domiciliary products used.

Using the 4.3× prismatic magnification technology, the etiological factor over gingival tissue inflammation was removed. The clinical method D-BioTECH, which allows to realize a deplaquing and a debridement above and below minimally invasive gingival, was performed [14].

During these maneuvers, a high-volume suction cannula with an integrated mirror was used (Purevac HVE), which ensures a greater reduction of fluids, optimized acoustics, and a decrease of up to 90% of aerosols and splatter compared to a traditional saliva vacuum (T1) [22, 23].

After a further 7 days, before continuing with the instrumentation under gingival, the patient's motivation was strengthened. Plaque detectors were used to share biofilm with the patient using photographic images. The instrumentation under gingival in full mouth (FM) regime in 2 days was performed. Ultrasonic inserts of different shapes were used, and some periodontal pockets were also equipped with mini-five curettes (T2).

After 21 days, the dental team received the patient for a motivational session and D-biotech (T3).

To strengthen the motivation and engraftment of probiotics in the oral cavity, the patient after another 21 days was reviewed, where the clinical approach D-BioTECH was used (T4).

**Figure 5:**
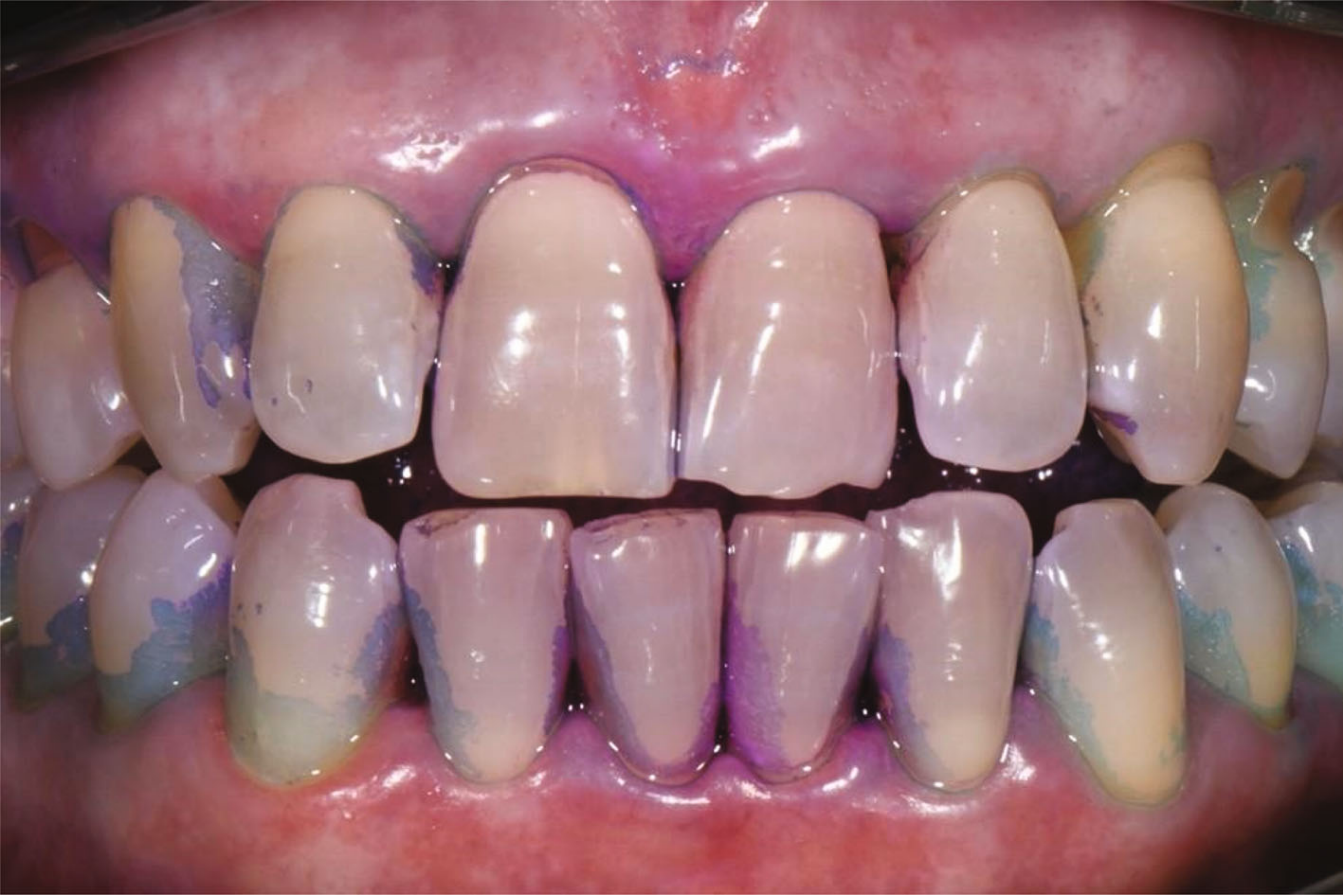
Front sector with plaque detector. First deplaquing.

**Figure 6:**
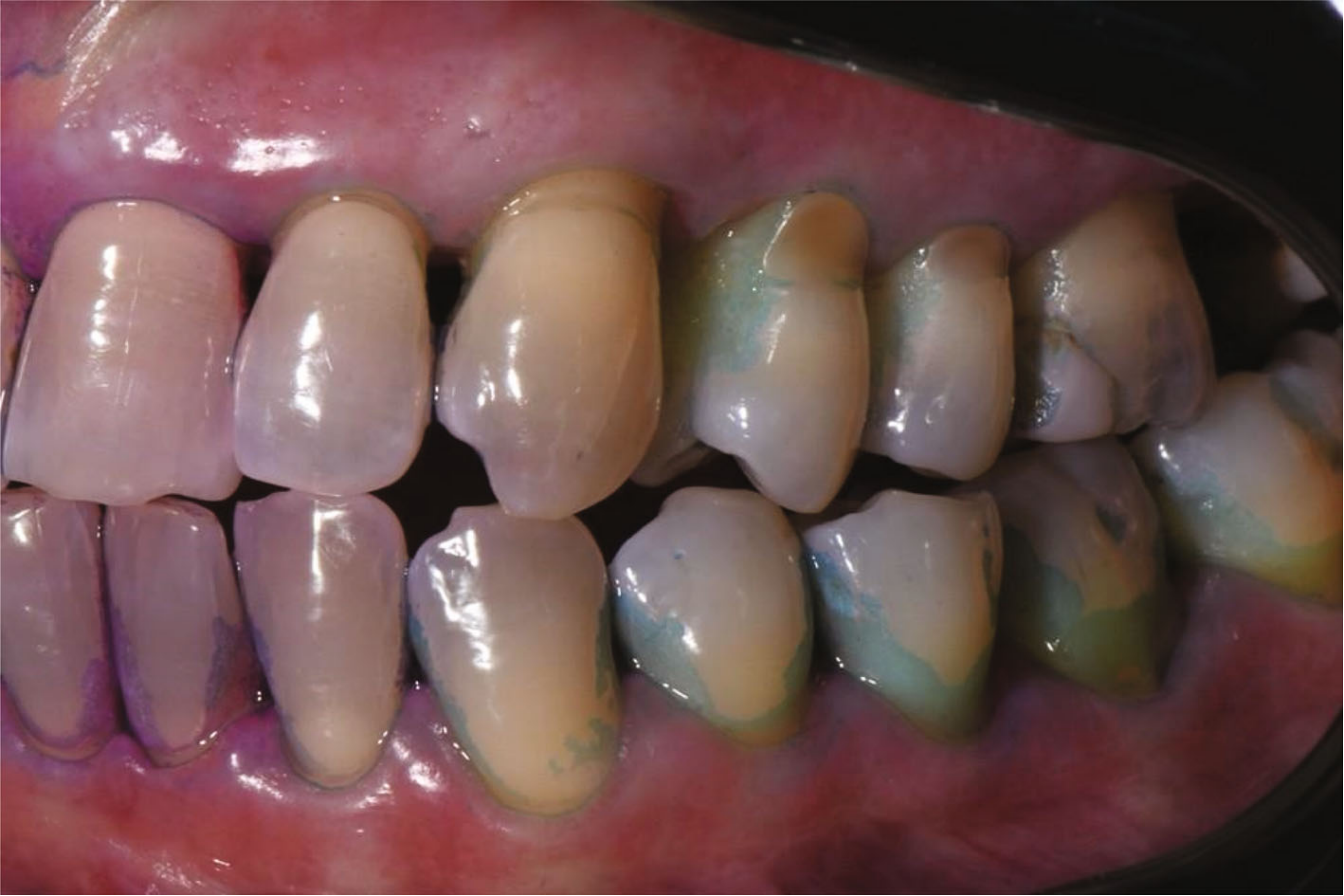
Left rear sector with plaque detector. First deplaquing.

**Figure 7:**
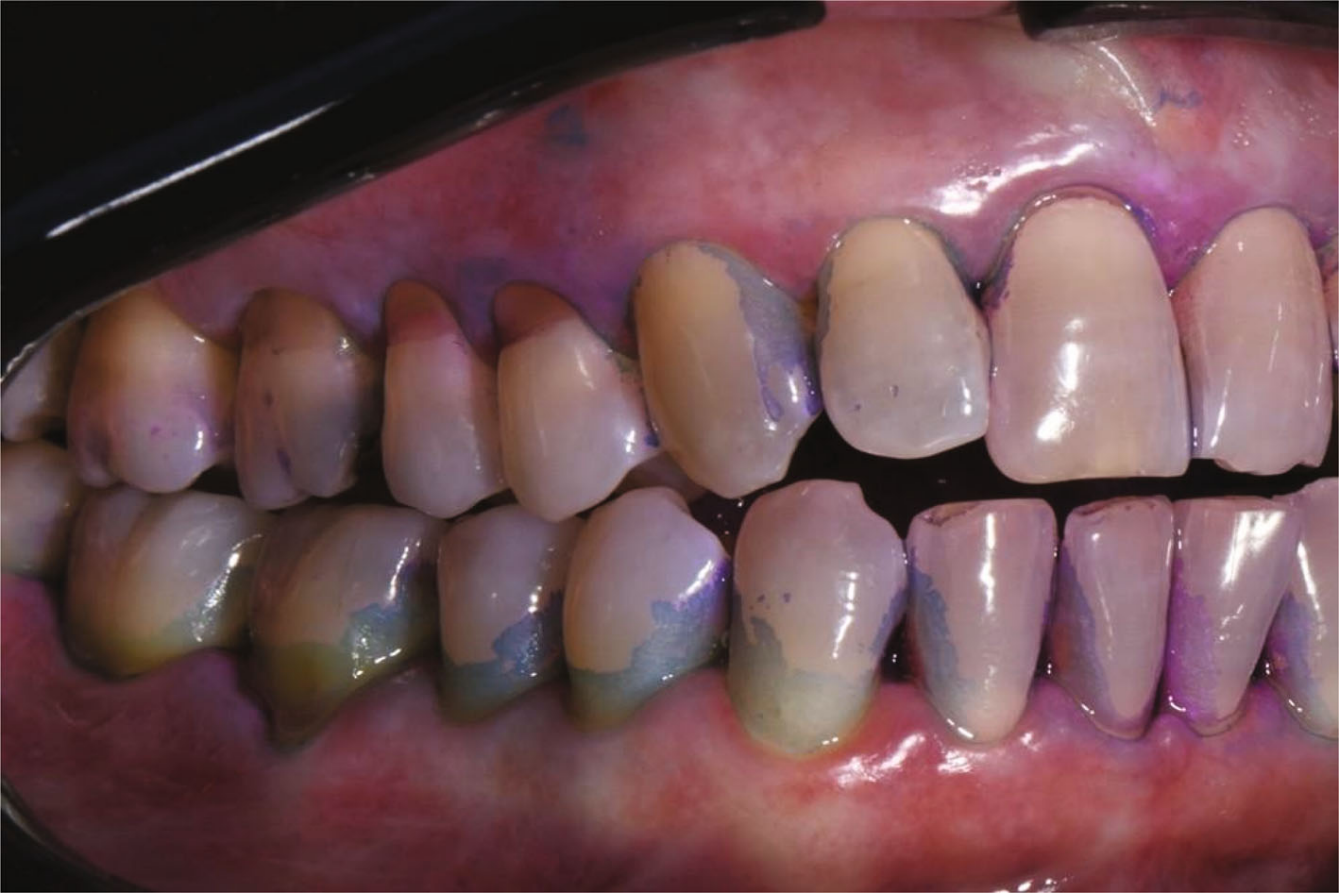
Right rear sector with plaque detector. First deplaquing.

Figures 5, 6, and 7 show photos of the patient at first deplaquing.

## 3. Results

After 60 days from the instrumentation under gingival, dental team received the patient for re-evaluation and find that the clinical parameters were significantly improved. In fact, it has gone from 82 to 14 total pockets with PPD equal to or greater than 4 mm and from 50 to 2 pockets with PPD equal to or greater than 5 mm. The FMBS rose from 48% to 15% and the FMPS from 73% to 15%. The results obtained fully meet the objectives of the periodontal protocol (T5).

After a further 60 days from the revaluation, dental team received the patient for a maintenance periodontal therapy session (with D-BioTECH approach). The two periodontal pockets that were still with PPD equal or greater than 5 mm (3.6 and 4.7 disto-lingual) were surveyed, and an improvement was observed with a 3 mm PPD. The dental hygienist insisted on strengthening the patient's motivation, especially in the area between 3.1 and 4.1.

Finally, the patient was reviewed for the protocol of home maintenance, reducing the use of mouthwash to 1 time a day (in the evening) and suspending the use of chewable gum [20].

The patient has reached all targets and was included in the maintenance periodontal protocol with quarterly recall (T6).

**Figure 8:**
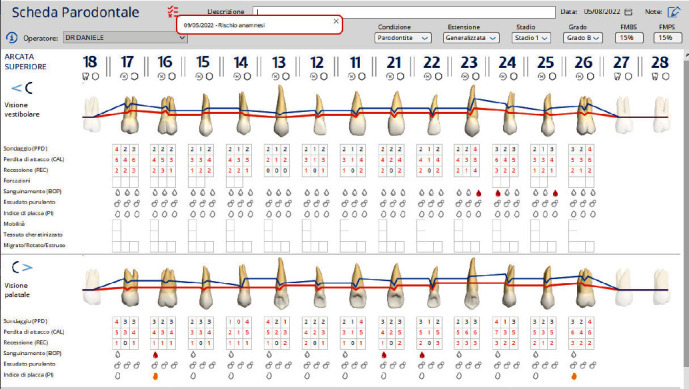
Revaluation to 60 days. Upper arch.

**Figure 9:**
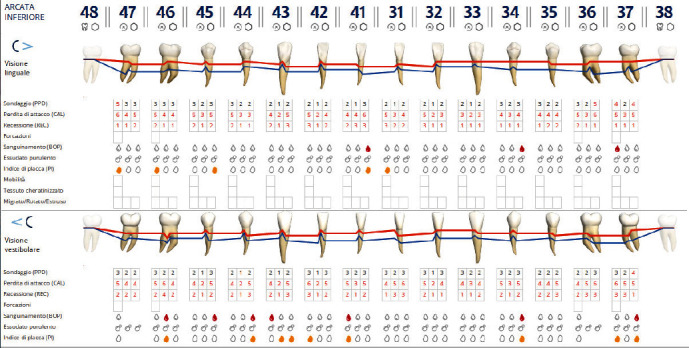
Revaluation to 60 days. Lower arch.

**Figure 10:**
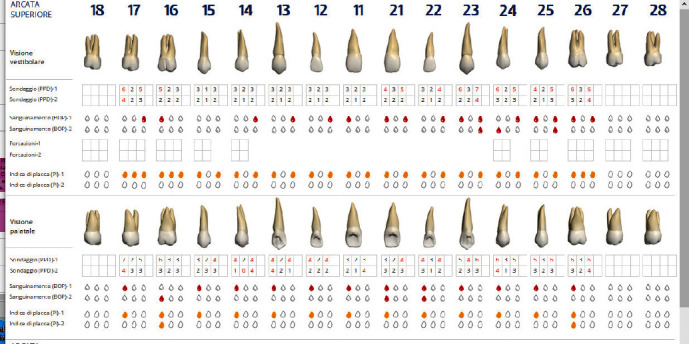
Comparison charting at 60 gg. Upper arch.

**Figure 11:**
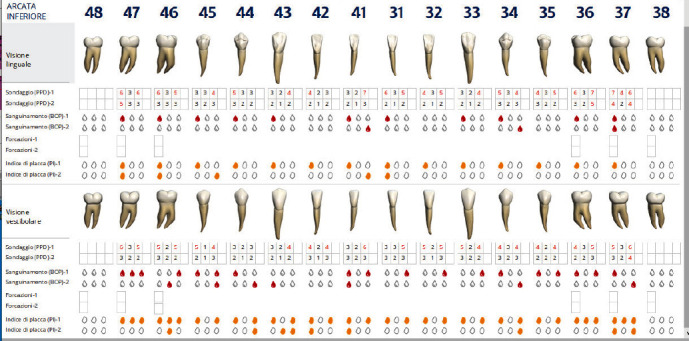
Comparison charting at 60 gg. Lower arch.

**Figure 12:**
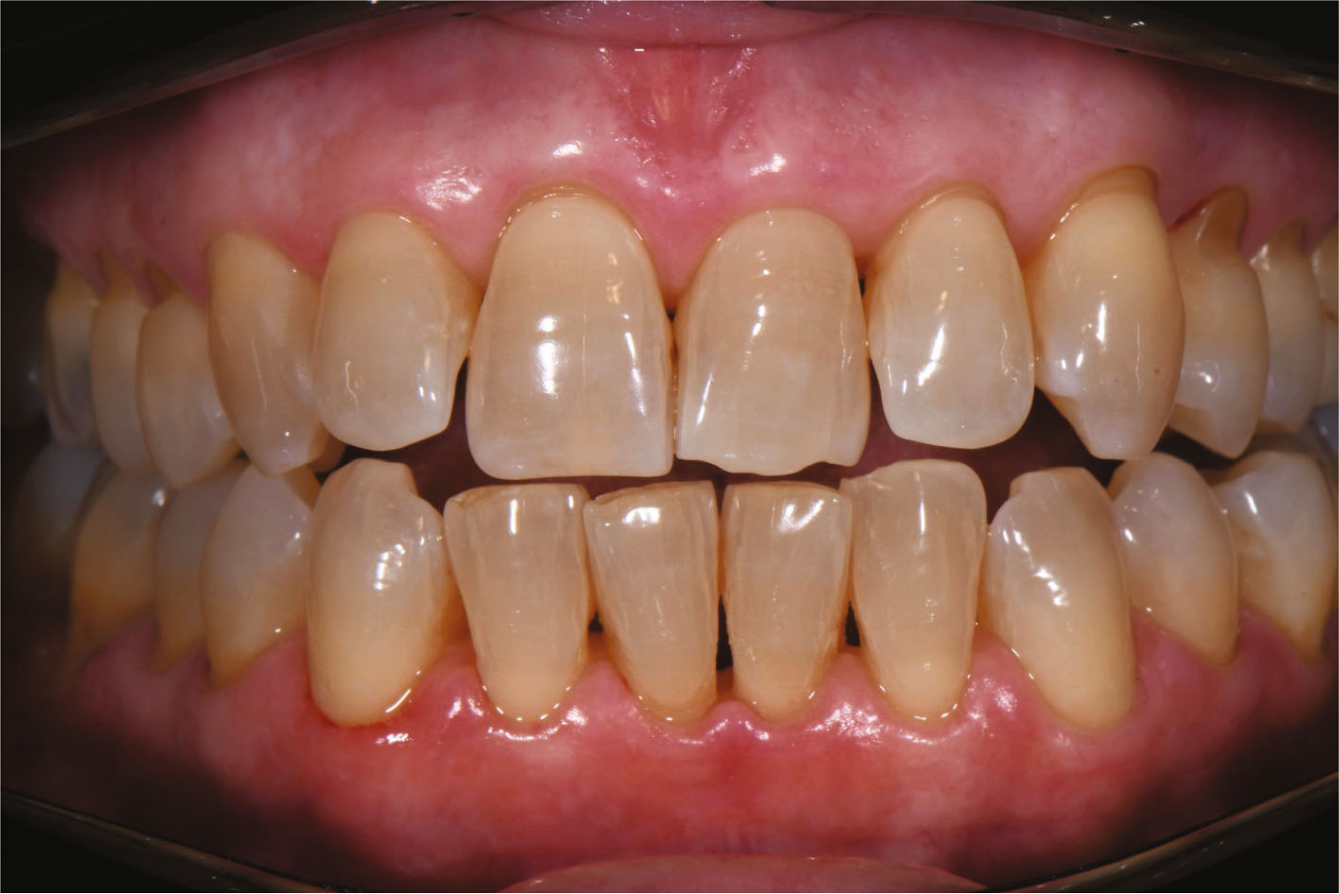
Results at 60 days. Front sector.

**Figure 13:**
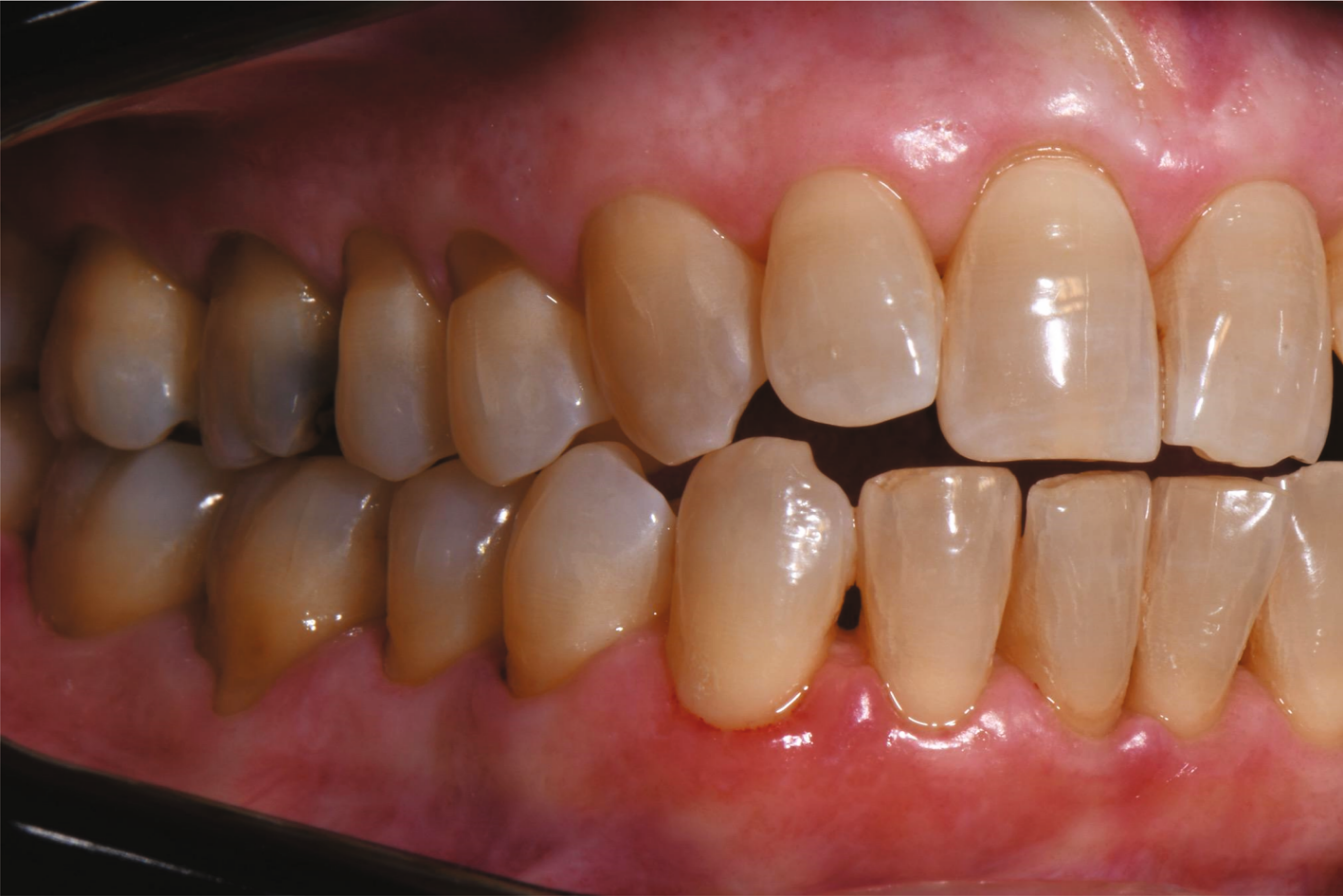
Results at 60 days. Right sector.

**Figure 14:**
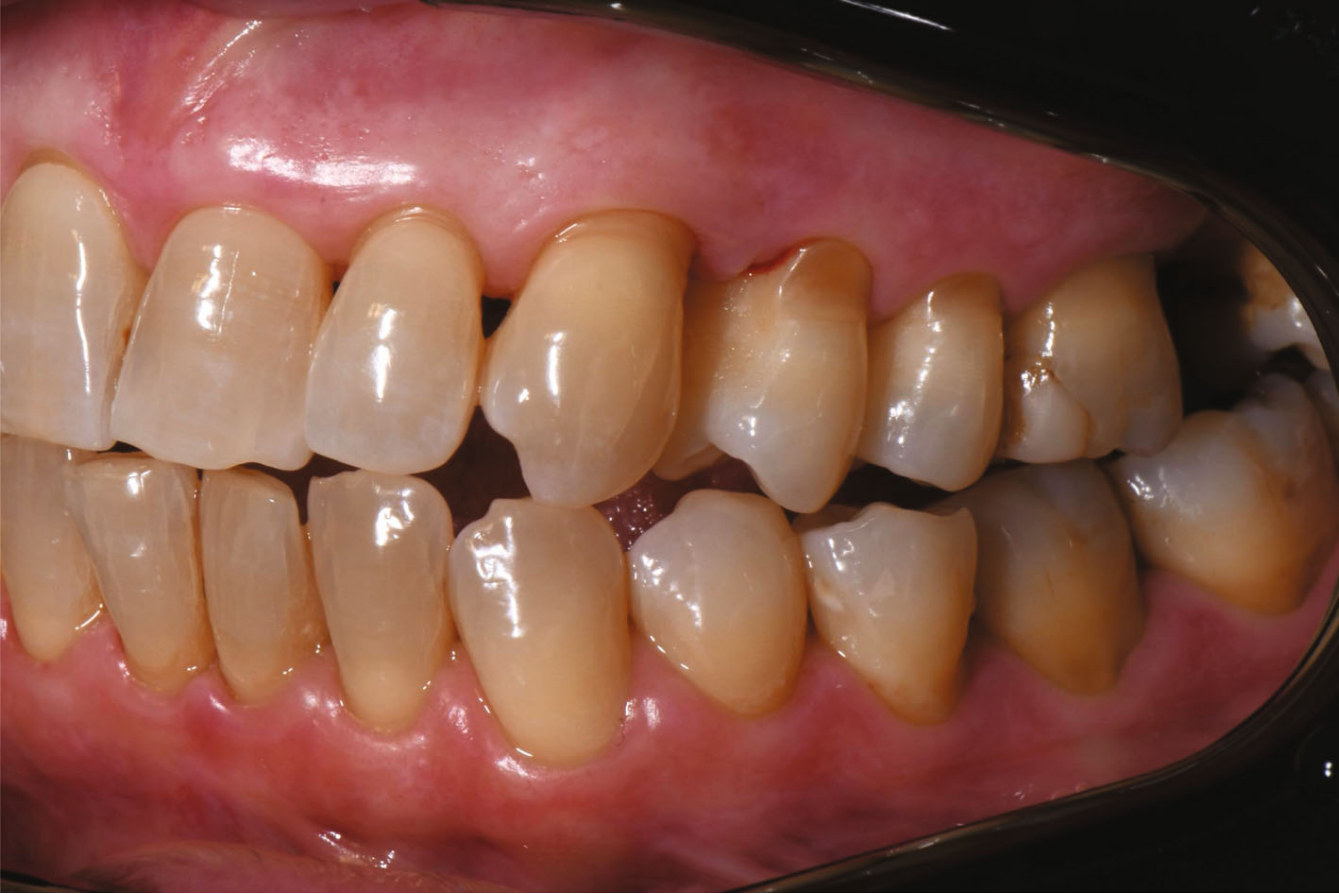
Results at 60 days. Left sector.

Figures 8, 9, 10, and 11 show periodontal charting after 60 days. Figures 12, 13, and 14 show photos of the patient at the end of the follow-up.

## 4. Discussion

In patients of Mc Ardle, muscle glycogen-phosphorylase does not work: during exercise, the muscle cells of patients deplete all their energy and are not able to produce more, when cells are unable to transform stored glycogen into glucose. In a short time, the lack of glucose causes fatigue and muscle stiffness during physical activity, bringing pain, and muscle damage [1, 15, 24].

The typical symptom is pain caused by anaerobic exercise, then repetitive movements, rapid movements, and maintaining posture; the pain begins a few minutes after exercise. From the literature, it emerged that physical weakness and intolerance to exercise are greater in women, even in carrying out the most common daily activities: it emerged that some patients had to use an electric toothbrush because they were easily tired brushing their teeth with a common manual toothbrush; in fact, muscle weakness of the arms is common [17, 25].

It can, therefore, be assumed that having fatigue and muscle stiffness in the arms, the oral hygiene of McArdle patients could be not optimal. Poor oral hygiene increases the risk of periodontal disease; those who regularly brush their teeth are less likely to develop periodontal disease [26]. It is not intended in this case that there is an association between McArdle's disease and periodontal disease, but since poor oral hygiene is known to be a risk factor for periodontitis, it can be assumed that patients with McArdle's disease may have periodontal problems. Unfortunately, there are no scientific studies in the literature, especially regarding this type of glycogenosis, unlike others already related to periodontal problems [5, 6].

Recent research showed the importance of probiotics in various dental fields. Latest trends divide these compounds into paraprobiotics (heat-inactivated bacteria) [20], lysates (bacterial fragments) [27], and postbiotics (concentrated bacterial active metabolites) [28], and all showed promising results in clinical dentistry.

Future research is needed to improve current knowledge about all these treatment possibilities.

The results of the present report are in agreement with the scientific literature present, concerning the effectiveness of the use of probiotics in non-surgical periodontal therapy: a study on patients with chronic periodontitis, to whom two tablets of probiotics were prescribed (*Lactobacillus reuteri*) following scaling and root planing, showed a significant reduction of anaerobic microorganisms in the biofilm subgingival [12]. Similar is a study conducted, which proposed to analyze patients with generalized periodontitis, undergoing non-surgical periodontal therapy together with two tablets of probiotics per day (*Bifidobacterium animalis* subsp. *lactis* HN019) or a placebo. They were then reassessed to 30 and 90 days from T0. A significant decrease in PPD was found in the test group (with higher gain in Clinical Attachment Level (CAL)), a decrease in red and orange complex periodontal pathogenic bacteria, and a decrease in proinflammatory cytokine levels. In addition, an increase in the number of *B. lactis* HN019 DNA was found in the subgingival biofilm [29].

Other studies have shown that Bifidobacterium can reduce the number of *Porphyromonas gingivalis* [30] and results similar to those just mentioned [31–36]. Although there have been promising clinical results, it would be necessary to conduct future studies to assess the effectiveness of probiotics in the long term, in the maintenance of periodontal patients, and especially in systemically compromised patients [37, 38]. Additionally, other recently introduced protocols, involving the use of ozone [39], lasers [40], or platelet-rich fibrin [41], could be tested in future trials.

## 5. Conclusions

This case is the first to assess the oral health of a patient suffering from McArdle's disease, a disease that can make the course of periodontal disease even more complex due to the difficulties in the maneuvers of oral hygiene at home.

In this case, the use of a “TBM” combined with the D-BioTECH has made it possible to considerably reduce the disease state, together with the use of probiotics at home to restore and maintain a healthy oral microbiome.

## Figures and Tables

**Table 1:** Composition of the domiciliary products.

Product	Composition
Toothpaste	Aqua, zinc hydroxyapatite∗, sorbitol, glycerin, hydrated silica, silica, cocamidopropyl betaine, cellulose gum, aroma, *Pistacia lentiscus* (mastic) gum oil, ascorbic acid, tocopheryl acetate, retinyl palmitate, sodium hyaluronate, *Hamamelis virginiana* leaf extract, spirulina platensis extract, *Calendula officinalis* flower extract, *Eucalyptus globulus* leaf oil, Bifidobacterium∗, Lactobacillus∗, sodium myristoyl sarcosinate, sodium methyl cocoyl taurate, phenoxyethanol, benzyl alcohol, sodium benzoate, sodium saccharin, potassium sorbate, maltodextrin, citric acid, *Helianthus annuus* seed oil, Butylhydroxytoluene (BHT), limonene, eugenol, CI 77891, CI 73360. ∗MicroRepairBioma.
Mouthwash	Aqua, sorbitol, xylitol, zinc hydroxyapatite∗, aroma, *Pistacia lentiscus* (mastic) gum oil, Lactobacillus∗, Bifidobacterium∗, sodium hyaluronate, ascorbic acid, *Hamamelis virginiana* leaf extract, spirulina platensis extract, *Calendula officinalis* flower extract, tocopheryl acetate, retinyl palmitate, *Eucalyptus globulus* leaf oil, PEG-40 hydrogenated castor oil, phenoxyethanol, sodium benzoate, cocamidopropyl betaine, glycerin, maltodextrin, sodium saccharin, *Helianthus annuus* seed oil, potassium sorbate, BHT, limonene, CI 16255. ∗MicroRepairBioma.
Chewable gum	Flavorings; emulsifier: soy lecithin; sweeteners: acesulfame, sucralose; antioxidant: tocopherols); bulking agents: isomalt, sorbitol; microRepair (orthophosphoric acid calcium salts); probiotic lactic ferments: *Lactobacillus reuteri* (SGL 01), *Lactobacillus salivarius* (SGL 03), *Lactobacillus plantarum* (SGL 07); support: maize maltodextrin; anti-caking agent: silicon dioxide, flavorings, vitamin C (calcium ascorbate), food coloring substances (radish and sweet potato concentrate); sweeteners: sucralose, acesulfame K; vitamin D (cholecalciferol).

∗: commercial name.

## Data Availability

The authors confirm that the data supporting the findings of this study are available within the article.
